# Antiviral Activity of Ag_5_IO_6_, a Unique Silver Compound

**DOI:** 10.3390/v16060959

**Published:** 2024-06-13

**Authors:** Mauri Erickson, Tyler J. Boone, Patricia L. Nadworny

**Affiliations:** 1 Nelson Laboratories Bozeman, LLC, 1765 S. 19th Avenue, Bozeman, MT 59718, USA; merickson@nelsonlabs.com; 2 Innovotech, Inc., Suite L131, 2011—94 St. NW, Edmonton, AL T6N 1H1, Canada; tyler.boone@innovotech.ca

**Keywords:** silver periodate, nanostructured silver, antiviral, antimicrobial coating, COVID-19, antibiofilm, anti-adherence, pentasilver hexaoxoiodate, InnovoSIL™-1 silver, virucidal

## Abstract

Pentasilver hexaoxoiodate (Ag_5_IO_6_) has broad-spectrum antimicrobial efficacy, including the long-term prevention of microbial adherence, the rapid killing of planktonic microorganisms, and the elimination of mature biofilms. This study’s goal was to determine whether it may also have antiviral activity against structurally distinct viruses. Ag_5_IO_6_ was tested following ASTM E1052-20, Standard Practice to Assess the Activity of Microbicides Against Viruses in Suspension, against adenovirus type 5, murine norovirus, poliovirus type 1, SARS-CoV-2 (original), and SARS-CoV-2 (omicron) (host cells: H1HeLa, RAW 264.7, LLC-MK2, Vero E6, and Vero E6, respectively). A 0.1 g/mL Ag_5_IO_6_ suspension was prepared and the viruses were exposed for 30 min, 4 h, or 24 h. Exposure to Ag_5_IO_6_ resulted in complete kill of SARS-CoV-2 (omicron) within 30 min, as well as complete kill of both SARS-CoV-2 (original) and the murine norovirus within 4 h. Ag_5_IO_6_ showed increasing activity over time against the adenovirus, but did not achieve a 3-log reduction within 24 h, and showed no antiviral activity against the poliovirus. These results demonstrate that Ag_5_IO_6_ has antiviral activity against medically important viruses, in addition to its well-characterized antimicrobial activity, suggesting that it may be valuable in situations where the prevention or simultaneous treatment of microbes and viruses are necessary.

## 1. Introduction

The development of antimicrobial resistance (AMR) is an ever-increasing public health problem, threatening humanity’s ability to effectively treat a broad range of bacterial, fungal, viral, and parasitic infections [1]. Various silver-containing products have been developed in the hope that they can be used as an antibiotic alternate to address concerns regarding increases in antimicrobial resistance combined with the narrow range of activity of some antibiotics. While it is well established that oxidized silver species have antimicrobial properties [2,3], the use of silver in clinical settings has been hampered because Ag^+^, typically the active species, is readily inactivated by contact with various substances, including bodily fluid components such as chloride ions and proteins [4]. In addition, silver-containing compounds are also often challenging to incorporate into/coat onto surfaces and devices with appropriate release kinetics for the intended applications, and may have limited activity, either generally [5,6] or against biofilms [7,8]. Moreover, limited testing has been carried out to determine whether they have antiviral properties. 

Ag_5_IO_6_ was originally developed for electrochemical cell applications [9], but has since demonstrated efficacy against both planktonic and preformed biofilm phenotypes of a broad range of microorganisms, as well as an ability to prevent microbial adherence to a variety of wound dressing surfaces [8]. The efficacy of Ag_5_IO_6_ in a simulated wound environment, via dissolution from coated wound dressing surfaces, where many other silver antimicrobial compounds are readily inactivated, is likely due to silver being present in both the cation, as [Ag_3_^3+^] which is anticipated to dissociate as Ag^+^, and the anion, as [Ag_2_IO_6_]^3−^ [10], and is enhanced by a synthesis method that combines nanoscale-sized grains into polycrystalline microscale-sized particles, such that the final product contains micron-sized particles with nanostructured features on them [11]. Its efficacy in complex media, along with appropriate release characteristics when it is incorporated into/coated onto a variety of surfaces (including medical-grade polymers [12], metals [13,14], a variety of wound dressing materials [8,14,15], and gels [16]), suggests that Ag_5_IO_6_ may be an effective tool to reduce antibiotic use by using it in applications to prevent microbial spread. Table 1 provides a brief comparison of Ag_5_IO_6_ with silver nanoparticles and typical ionic silver treatments such as AgNO_3_ and AgCl.

There are limited data demonstrating antiviral activity for oxidized silver species. However, a recent study showed that solutions generated from a nanostructured silver metal/silver oxide wound dressing had antiviral activity against herpes simplex virus 1 (HSV-1) and severe acute respiratory syndrome coronavirus 2 (SARS-CoV-2—original strain) in vitro, which translated to rapid symptom remission in non-hospitalized patients with coronavirus disease of2019 (COVID-19) [17]. In the current study, it was hypothesized that Ag_5_IO_6_ would also have antiviral activity, given that it uses similar mechanisms of action to the antiviral nanosilver-derived solutions described above [17]. 

The purpose of this work was to determine if Ag_5_IO_6_ also has antiviral properties, which would improve its usefulness in medical device coatings, as well as increasing its potential applications, for example, to high-touch surfaces and various air filters. This study evaluated the virucidal efficacy of Ag_5_IO_6_ when challenged with viral suspensions of adenovirus type 5; murine norovirus; poliovirus type 1; and two SARS-CoV-2 isolates in vitro, using a time-kill method with 30 min, 4 h, and 24 h challenges based on ASTM E1052-20 [26], under clean conditions. Ag_5_IO_6_ was able to rapidly inactivate the norovirus and two SARS-CoV-2 strains, while there was minimal activity against the adenovirus, and no activity against the poliovirus. 

## 2. Materials and Methods

### 2.1. Materials

Ag_5_IO_6_ was synthesized, following a variation of Nadworny et al. [11], by ChemForce Laboratories, Inc., Edmonton, AB, Canada (lot CF-011-055). Product purity was determined just prior to antiviral testing via X-ray diffraction at the Department of Earth, Ocean, and Atmospheric Sciences, University of British Columbia. It was 96.6% Ag_5_IO_6_ (remainder silver metal), with a grain size of 19.1 ± 0.5 nm. For the challenge, 0.01 g/mL Ag_5_IO_6_ was added to distilled water and slurried by stirring for approximately 4 h at room temperature. The suspension thus created was mixed well prior to dispensing for the challenge.

Media used included 1× Minimum Essential Medium (MEM) or other appropriate medium such as advanced MEM, EMEM (Eagle’s Minimal Essential Medium, ATCC), DMEM (Dulbecco’s Modified Eagle Medium, ATCC), or RPMI-1640 (Roswell Park Memorial Institute medium, ATCC); growth medium (GM): MEM or other medium with 4–10% serum, 1% antibiotic/antimycotic, and 1% L-glutamine (when necessary, Gibco, Grand Island, USA); maintenance medium (MM): MEM or other media with 2–10% serum, 1% antibiotic/antimycotic, and 1% L-glutamine (when necessary); and Trypsin/EDTA (ethylenediaminetetraacetic acid, ATCC). The antibiotic/antimycotic used contained 10,000 units/mL penicillin, 10,000 µg/mL streptomycin, and 25 µg/mL amphotericin B. The serum used was fetal bovine serum or horse serum. The neutralizer used was Dey-Engley (D/E) neutralizing broth.

### 2.2. Challenge Viral Strains and Host Cells

The following challenge viral strains were used: Adenovirus type 5, strain Adenoid 75 (ATCC #VR-5—incubation time: 5–10 days); murine norovirus strain S99 (Friedrich-Loeffler Institute, Greifswald, Germany, #RVB-651—incubation time: 5–8 days); poliovirus type 1 strain Chat (ATCC #VR-1562—incubation time: 5–7 days); SARS-CoV-2 isolate USA-WA1/2020 (BEI Resources #NR-52281, referred to as “original” throughout—incubation time: 9–14 days); and SARS-CoV-2 isolate hCoV-19/USA/MD-HP20874/2021 (BEI Resources #NR-56461—referred to as “omicron” throughout—incubation time: 9–14 days), where ATCC is the American Type Culture Collection, and BEI Resources refers to the Biological and Emerging Infections Resources Program of the National Institute of Allergy and Infectious Diseases (NIAID). The test virus suspensions used were from high-titer stock, propagated and stored at approximately −70 °C following standard procedures [26], with incubation at ~37 °C. Aliquots were thawed on the day of use.

Host cells were maintained as monolayers in disposable cell culture labware. Prior to testing, host cell cultures were seeded onto multi-well cell culture treated plates. Cell monolayers were <48 h old when used in testing. H1HeLa cells (ATCC #CRL-1958; human cervical epithelial adenocarcinoma) were approximately 90% confluent prior to inoculation with adenovirus type 5. RAW 264.7 cells (ATCC #TIB-71; mouse macrophage) were approximately 80% confluent prior to inoculation with murine norovirus. LLC-MK2 cells (ATCC #CCl-7.1; rhesus monkey kidney epithelial cells) were approximately 80% confluent before inoculation with poliovirus type 1. Vero E6 cells (ATCC #CRL-1586; green monkey kidney cells, epithelial) were approximately 80% confluent before inoculation with either SARS-CoV-2 strain. At this point, growth medium (GM) was replaced by maintenance medium (MM) to support virus propagation.

For the test article, 0.5 mL aliquots of test virus were added to vials containing 4.5 mL of the test article for the selected exposure time in a refrigerator, followed by neutralization, dilution, and plating. Exposure was performed refrigerated to ensure minimum virus titers were met by reducing the die-off of viruses in the controls during longer exposure times for viruses that are temperature-sensitive. All samples involving the test article were periodically inverted to support the slurrying/suspension of the test article. Virus controls were performed by combining 0.5 mL aliquots of test virus with 4.5 mL of MM for the selected exposure period in a refrigerator, followed by dilution and plating. For cytotoxicity controls, 0.5 mL aliquots of MM were added to the test article, followed by neutralization, dilution, and plating. To confirm neutralizer efficacy, 0.5 mL aliquots of MM were added to vials containing 4.5 mL of the test article, and added to neutralizer prior to virus inoculation, exposure for at least the selected exposure time in the refrigerator, dilution and plating. To ensure that there was no neutralizer toxicity, 0.5 mL aliquots of each virus was added to 4.5 mL of the neutralizer, and exposed for at least the selected exposure time in the refrigerator, followed by dilution and plating. The difference in virus titer for the neutralization control, neutralizer toxicity control, and virus control did not exceed 1.0 log_10_, indicating that neutralization was effective and the neutralizer was nontoxic. The intact cell culture served as the control of cell culture viability, replacing GM with MM. For each test parameter, samples were centrifuged at 1500 rpm for 5 min, to remove any remaining test article precipitate, prior to 10-fold dilutions of the supernatant in MM and plating. Each dilution was plated in 4 replicates in 1.0 mL aliquots per well. The virus was allowed to adsorb for approximately 1–2 h in a 37 ± 2 °C CO_2_ incubator before the test dilutions were removed from the plates and replaced with media. Plates were incubated in a CO_2_ incubator under appropriate growth conditions as described above. Cytopathic/cytotoxic effects were monitored using an inverted compound microscope. 

Viral and toxicity titers are expressed as -log_10_ of the 50% titration end point for infectivity. Calculations of the estimated virus concentrations were performed using a 50% tissue culture infectious dose (TCID_50_) calculation, applying the Quantal test (Spearman–Kärber Method [27]):

log_10_ TCID_50_ = L − d (s − 0.5)

where:

L = −log_10_ of the lowest dilution;

d = difference between dilution steps;

s = sum of proportions of positive wells.

The log_10_ of infectivity reductions were calculated as follows:

log_10_ Reduction = (log_10_ TCID_50_ of the Virus Control) − (log_10_ TCID_50_ of the Virucidal Suspension Test)

Percent reductions were calculated as follows:

% Reduction = [1 − (TCID_50_ test)/(TCID_50_ virus control)] × 100

## 3. Results

Details of the results obtained after the 30 min, 4 h, and 24 h exposures for adenovirus type 5, murine norovirus, poliovirus type 1, and SARS-CoV-2 original and omicron strains are available in the Supplementary Material as Tables S1–S15. It should be noted that the cell controls consistently had no virus growth in any of the four replicates for all tests performed. Test replicates were compared to virus controls for the determination of log reductions. Cytotoxicity from Ag_5_IO_6_ was only observed at the −2 dilution, except in the LLC-MK2 cells used with poliovirus, where it was not observed, and did not impact study outcomes. Neutralizer control and neutralizer cytotoxicity control data were consistently comparable to the virus control data, indicating that the neutralizer used was effective and did not cause cytotoxicity.

Table 2 provides a summary of the results from all time points tested. As shown in Table 2, Ag_5_IO_6_ reduced the infectivity of adenovirus type 5 by 0.4 log_10_ following a 30 min exposure, slightly less than 1 log_10_ following a 4 h exposure, and 1 log_10_ following a 24 h exposure. Although increasing activity was observed with time, Ag_5_IO_6_ did not demonstrate ≥3 log_10_ reduction of adenovirus type 5 at any of the tested time points. Ag_5_IO_6_ reduced the infectivity of murine norovirus by slightly less than 3 log_10_ following a 30 min exposure, and 5 log_10_ following 4 and 24 h exposures, with complete elimination at the latter two time points, within the limits of detection of the study. Ag_5_IO_6_ reduced the infectivity of poliovirus type 1 by <1 log_10_ at all exposure times, with no trends towards increasing activity with time. Ag_5_IO_6_ reduced the infectivity of the original SARS-CoV-2 strain by slightly less than 2 log_10_ after the 30 min exposure, and ≥3 log_10_ after 4 and 24 h exposures, with complete elimination at the latter time point, within the limits of detection of the study. Similarly, Ag_5_IO_6_ reduced the infectivity of the SARS-CoV-2 omicron variant by ≥3 log_10_ at all tested time points, with complete elimination, within the limits of detection of the study.

## 4. Discussion

The results of this study demonstrate that Ag_5_IO_6_ has virucidal activity against norovirus, as well as two different SARS-CoV-2 strains. Compared to the earlier study of nanosilver-derived solutions, which required 24 h to achieve the complete killing of the original SARS-CoV-2 strain [17], Ag_5_IO_6_ generated total killing within 4 h for the original SARS-CoV-2 strain, and within 30 min for the omicron strain, despite both agents utilizing similar modes of action (i.e., the oxidation of essential viral proteins) [28]. This is similar to previous work demonstrating the increased antimicrobial activity of Ag_5_IO_6_ relative to nanostructured silver/silver oxide dressings [8]. The difference observed may be due in part to the combination of silver with high oxidation state iodine, as studies have shown that certain forms of iodine are virucidal (e.g., Naqvi et al. [29], Chopra et al. [30]), and therefore the combination of iodine and silver in Ag_5_IO_6_ may synergistically improve its antiviral activity. It is interesting to note that the omicron strain appears to be more susceptible to Ag_5_IO_6_ than the original SARS-CoV-2 strain. Future work could compare these viruses and the effect of Ag_5_IO_6_ on them to determine the cause of the improved target viruses’ susceptibility, which could help to identify specific viral targets on which Ag_5_IO_6_ acts.

This study demonstrates that Ag_5_IO_6_ had in vitro efficacy against certain viruses (SARS-CoV-2 and norovirus), but not against others (adenovirus and poliovirus). Its activity against both the original and omicron strains of SARS-CoV-2 suggests that it should be effective against both current and future variants of COVID-19, with potential for activity against other epithelium-based viral infections too. SARS-CoV-2 is an enveloped, positive-sense, single-stranded RNA virus typically causing acute respiratory illness. Given that in the study of nanosilver solutions, a > 3 log reduction was seen against HSV-1 within 3–4 h of challenge [17], and given that at least some mechanisms of action for antiviral activity are anticipated to be in common between the nanosilver solutions and the Ag_5_IO_6_ slurry, this suggests that Ag_5_IO_6_ would also be rapidly efficacious against HSV-1, which is an enveloped, double-stranded DNA virus causing oral herpes. It is anticipated that, based on these results, Ag_5_IO_6_ is likely to show efficacy against influenza and respiratory syncytial viruses (RSVs) as well, as they are also respiratory, enveloped, negative-sense, single-stranded RNA viruses. Both of these viruses are known to be sensitive to iodine, and Ag(I) could have several targets, including blocking virus-cell binding in enveloped viruses in general [31].

Non-enveloped viruses are generally more resistant to a wide variety of disinfectants than enveloped viruses because the lipid envelope is easier to disrupt than the protein capsid in non-enveloped viruses [32]. One of the known mechanisms of action for silver antimicrobial activity is targeting cell membranes [33], suggesting that a similar mechanism may contribute to the antiviral activity of Ag_5_IO_6_. However, Ag_5_IO_6_ was also active against norovirus, which is a non-enveloped, positive-sense, single-stranded RNA virus causing acute gastroenteritis, suggesting an additional mode of action. Ag_5_IO_6_ was found to be less effective against two other non-enveloped viruses—adenovirus type 5, which causes respiratory illness, and poliovirus, a serious infection of the nervous system. The lack of activity in these viruses suggests that Ag_5_IO_6_ may not be able to bind to the capsid of these viruses in a way that would prevent infection. Interestingly, the test article/neutralizer suspension did not have a significant effect on the LLC-MK2 host cell line, which was used to propagate poliovirus, but did show some cytotoxicity to the other cell lines. It should be noted that cytotoxicity in mammalian cell culture lines does not correlate with in vivo safety results—virtually all silvers releasing sufficient species for antimicrobial activity fail standard in vitro cytotoxicity testing, partly due to the absence of a humoral response ex vivo, and thus require in vivo safety testing [34]. The demonstrated activity of Ag_5_IO_6_ against both enveloped and non-enveloped viruses suggests that it has multiple antiviral targets, with efficacy that is not based on one mechanism of action alone, suggesting that it may have activity against a wide variety of viruses. 

In microorganisms, silvers have a number of mechanisms of action, including the following: interactions between oxidized silver and structural proteins, enzymes, and various other components of cell membranes, particularly those with negatively charged thiol groups, allowing the silver to bind to and damage the cell membranes [33,35]; ionic silver acting as an oxidizing agent to support the production of reactive oxygen species (ROS), which impair metabolic processes and cell division [36]; ionic silver interfering with the respiratory chain, due at least partially to its strong interaction with thiol groups present in microbial respiratory enzymes and other electron transport chain components [28,37,38]; and ionic silver preferentially binding with amino groups of nucleic acids such as DNA bases, thus inhibiting replication [33,35]. Ag_5_IO_6_ is anticipated to act via all of the above mechanisms of action, since it acts via the release of silver-containing ions, noting that the Ag_5_IO_6_ does not contain or release silver nanoparticles. It is anticipated that the addition of iodine present in Ag_5_IO_6_ results in further mechanisms of action. The results presented in this work suggest that Ag_5_IO_6_ antiviral activity may also have multiple modes of action, potentially targeting both the virus particles and host cell machinery. The fact that Ag_5_IO_6_ has demonstrated multiple targets in this study, combined with potent activity, suggests a reduced probability of viruses developing resistance to it. 

Severe cases of COVID-19 result in inflammation and subsequent secondary microbial infections of patients’ lungs. Various treatments, such as Remdesivir, cortical steroids, and dexamethasone, have had limited success against these severe cases [39,40], while some antiviral drugs developed over the course of the pandemic had undesirable side effects and/or interactions with other medications [41]. New COVID variants were found to emerge rapidly, and vaccination has not consistently prevented infection by variants such as omicron, particularly when not recently boosted [42]. As such, more effective treatments and preventative measures are still needed, even as the pandemic winds down. Similar challenges have been found during the development of antivirals for other diseases, indicating a need for methods to reduce infection rates, and thus reduce associated hospitalization, mortalities, and other adverse events such as long-term poor health outcomes, with their associated costs. The main source of COVID-19 infection, and various other viral infections, is the transmission of droplets released during exhalation (including sneezes/coughs) by infectious individuals. Viral particles can remain in the air and on surfaces for extended periods of time [43]. To reduce the risk of transmission, high-touch public surfaces, hospital equipment, and certain medical devices are disinfected regularly/prior to use. Current protocols for disinfecting these surfaces using chemicals such as bleach have disadvantages, including cost, due to the disinfection frequency required; the release of irritant gases that can trigger asthma attacks and are linked to other respiratory conditions; and disposal requirements for containers after use [43]. The development of self-cleaning surfaces/antiviral surfaces with long-term stability/durability would address most, if not all, of the above concerns by repelling or neutralizing viruses. 

An increased awareness of and a desire to reduce viral transmission through the development of antiviral coatings have led to the question of whether any antimicrobial compounds could also be used to prevent viral spread. For example, incorporating antimicrobial and antiviral agents into coatings for high-touch surfaces and medical devices is expected to dramatically reduce the frequency of hospital-acquired infections, also reducing the resultant extended hospitalizations, mortalities, and long-term impacts on health. Some surface coating options under consideration [43] include the following: gold nanoparticles, which unfortunately are more cost-prohibitive than coating with silver, have lower antimicrobial activity, and have the potential for toxicity issues [44]; copper nanoparticles, which have less antimicrobial activity than silver due to lower redox potential [45]; titanium-based photocatalysts, which only provide line-of-sight activity, and typically require significant times under UV radiation for sufficient antimicrobial activity to be achieved, limiting their applications [43]; and silver nanoparticles—single crystals of silver of <100 nm (e.g., Jeremiah et al. [46]), however, there are concerns regarding silver nanoparticle cytotoxicity/biocompatibility [22,23], lack of efficacy against viruses [17] and microbes [19], and tendency to self-agglomerate [43]. The use of Ag_5_IO_6_ addresses many of the issues associated with these options. Its simple manufacturing method combined with low quantities required for activity make it a cost-competitive yet effective option; it is easy to apply to surfaces and there are no requirements such as line-of-sight light treatments; it is unusually stable compared to other silver compounds; and initial testing has not raised any red flags regarding toxicity/biocompatibility [15]. Potential disadvantages to the use of Ag_5_IO_6_ include that it is dark brown, limiting its application in situations where the dark color is undesirable; it may stain if used at higher concentrations; and, due to its relatively low solubility combined with its density, it may be challenging to use in situations where a liquid treatment (e.g., a spray application) would be most desirable. Further testing mimicking conditions of production and use for intended applications will be warranted to identify other limitations on its use. 

This study demonstrated that Ag_5_IO_6_ has antiviral activity that is relevant to clinically important viruses, in addition to its already-established antimicrobial and anti-biofilm efficacy. Given that at least some viruses can colonize preexisting biofilms [47] while others cover themselves in a biofilm-like extracellular matrix (ECM) containing carbohydrates and proteins [48,49], the ability of Ag_5_IO_6_ to penetrate biofilms, unlike other silvers [8] that are inactivated or repelled by ECM components, may give it an additional advantage as an antiviral agent. Ag_5_IO_6_ may be a valuable agent in situations where either infection prevention or simultaneous treatment of microbes and viruses are desirable. Future research, including further testing against other virus families in vitro, further development of methods to coat Ag_5_IO_6_ onto relevant surfaces, and subsequent in vivo testing for antiviral activity, is necessary to better elucidate the efficacy of Ag_5_IO_6_ as a coating to reduce viral transmission.

## 5. Patents

(Pending) 18/233,409, Antimicrobial Silver Iodates, filed 14 August 2023, USA.

## Figures and Tables

**Table 1:** Comparison of different categories of silver technologies.

Characteristic	Ag_5_IO_6_	Silver Nanoparticles	Standard Ionic Silver (e.g., AgNO_3_, AgCl, etc.)
Release Profile	Slow-release, equilibrating at sufficient levels for activity [8,16]	Minimal release of active species [17,18,19]	Typically either poorly soluble or released as bolus [6]
Interaction with Bodily Fluids	Self-limited—remains active in the presence of chloride ions, proteins, etc. [8,16]	Released active species (Ag^+^) react with components to form insoluble compounds [4]	Released active species react with components to form insoluble compounds [4]
Stability	Good stability under a number of conditions (light, heat, solvents, and sterilization) [16]	Stability issues (capping often required) [20,21]	Varies with compound, often poor (e.g., not light stable, challenges with sterilization)
Impact on Biofilms	Effective [8]	Not reported, anticipated to be minimal based on poor activity against planktonic microorganisms [17,18,19]	Ineffective [8]
Ease of Incorporation into Products	Relatively simple [12,13,14,15,16]	Challenging due to stability issues described above	Challenging due to release profile and stability issues, as described above
Toxicity Concerns	None known, based on internal testing [15]	Nanoparticle-related toxicity concerns [22,23]	Can cause inflammation, slow healing [24]
Overall Biocidal Activity	Strong [8]	Minimal [17,18,19,25]	Variable, negatively impacted by biological fluids, release profile, and stability issues [5,6,8]

**Table 2:** Summary of virus average log_10_ reductions achieved with Ag_5_IO_6._

Virus	Viral Structure	30 min	4 h	24 h
Adenovirus	Non-enveloped, double-stranded DNA	0.42	0.92	1.17
Norovirus	Non-enveloped, single-stranded RNA	2.83	≥4.50	≥4.75
Poliovirus	Non-enveloped, single-stranded RNA	≤0.75	≤0.75	≤0.50
SARS-CoV-2 (original)	Enveloped single-stranded RNA	1.92	≥3.17	≥3.00
SARS-CoV-2 (omicron)	Enveloped single-stranded RNA	≥2.92	≥3.25	≥3.00

## Data Availability

Data are contained within the article and Supplementary Material.

## Supplementary Materials

The following supporting information can be downloaded at https://www.mdpi.com/article/10.3390/v16060959/s1: Table S1: 30 min challenge: Adenovirus type 5; Table S2: 4 h challenge: Adenovirus type 5; Table S3: 24 h challenge: Adenovirus type 5; Table S4: 30 min challenge: Murine norovirus; Table S5: 4 h challenge: Murine norovirus; Table S6: 24 h challenge: Murine norovirus; Table S7: 30 min challenge: Poliovirus type 1; Table S8: 4 h challenge: Poliovirus type 1; Table S9: 24 h challenge: Poliovirus type 1; Table S10: 30 min challenge: SARS-CoV-2 (original); Table S11: 4 h challenge: SARS-CoV-2 (original); Table S12: 24 h challenge: SARS-CoV-2 (original); Table S13: 30 min challenge: SARS-CoV-2 (omicron); Table S14: 4 h challenge: SARS-CoV-2 (omicron); Table S15: 24 h challenge: SARS-CoV-2 (omicron).
