# Supplementary material for: Antiviral Activity of Ag5IO6, a Unique Silver Compound
Source: Viruses. 2024 Jun 13;16(6):959. doi: 10.3390/v16060959 (PMC11209601; doi:10.3390/v16060959)
Supplement: Supplementary file 1 [file viruses-16-00959-s001.zip › viruses-3038204-supplementary.pdf]

Supplementary Material for:

Antiviral Activity of Ag_5IO_6, A Unique Silver Compound

Mauri Erickson, Tyler J. Boone, and Patricia L. Nadworny

Table S1. 30-minute challenge: Adenovirus type 5. "+" indicates virus-infected cells were present, while "0" indicates virus-infected cells were not detected. CT indicates concentration at which cytotoxicity was present. NT indicates "not tested". CTC is the cytotoxicity control.

Dilution (-Log_{10})	Virus Control	Test Rep. 1	Test Rep. 2	Test Rep. 3	CTC
-2	NT	CT	CT	CT	CT
-3	NT	++++	++++	++++	0000
-4	++++	++++	++++	++++	0000
-5	++++	++++	++++	++++	NT
-6	++++	++++	++++	++++	NT
-7	++++	+00+	+0++	++00	NT
-8	0000	NT	NT	NT	NT
$TCID_{50}$/mL (log_{10})	7.50	7.00	7.25	7.00	2.50
Log_{10} Reduction	N/A	0.50	0.25	0.50	N/A
Percent Reduction	N/A	68.38%	43.77%	68.38%	N/A

Table S2. 4-hour challenge: Adenovirus type 5. "+" indicates virus-infected cells were present, while "0" indicates virus-infected cells were not detected. CT indicates concentration at which cytotoxicity was present. NT indicates "not tested". NC is the neutralizer control. NTC is the neutralizer cytotoxicity control. CTC is the cytotoxicity control.

Dilution (-Log_{10})	Virus Control	Test Rep. 1	Test Rep. 2	Test Rep. 3	NC	NTC	CTC
-2	NT	CT	CT	CT	NT	NT	CT
-3	NT	++++	++++	++++	++++	++++	0000
-4	++++	++++	++++	++++	++++	++++	0000
-5	++++	++++	++++	++++	++++	++++	NT
-6	++++	+0++	+0+0	++00	++++	++++	NT
-7	+00+	0000	0000	0000	0++0	0++0	NT
-8	0000	NT	NT	NT	0000	0000	NT
$TCID_{50}$/mL (log_{10})	7.00	6.25	6.00	6.00	7.00	7.00	2.50
Log_{10} Reduction	N/A	0.75	1.00	1.00	N/A	N/A	N/A
Percent Reduction	N/A	82.22%	90.00%	90.00%	N/A	N/A	N/A

Table S3. 24-hour challenge: Adenovirus type 5. "+" indicates virus-infected cells were present (n=4 per control or test replicate), while "0" indicates virus-infected cells were not detected. CT indicates concentration at which cytotoxicity was present. NT indicates "not tested". NC is the neutralizer control. NTC is the neutralizer cytotoxicity control. CTC is the cytotoxicity control.

Dilution (-Log10)	Virus Control	Test Rep. 1	Test Rep. 2	Test Rep. 3	NC	NTC	CTC
-2	NT	CT	CT	CT	NT	NT	CT
-3	NT	++++	++++	++++	++++	++++	0000
-4	++++	++++	++++	++++	++++	++++	0000
-5	++++	++++	++++	++++	++++	++++	NT
-6	++++	+00+	++0+	0+0+	++++	++++	NT
-7	++0+	0000	0000	0000	00++	0+0+	NT
-8	0000	NT	NT	NT	0000	0000	NT
$TCID_{50}$/mL (log_{10})	7.25	6.00	6.25	6.00	7.00	7.00	2.50
Log_{10} Reduction	N/A	1.25	1.00	1.25	N/A	N/A	N/A
Percent Reduction	N/A	94.38%	90.00%	94.38%	N/A	N/A	N/A

Table S4. 30-minute challenge: Murine norovirus. "+" indicates virus-infected cells were present, while "0" indicates virus-infected cells were not detected. CT indicates concentration at which cytotoxicity was present. NT indicates "not tested". CTC is the cytotoxicity control.

Dilution (-Log10)	Virus Control	Test Rep. 1	Test Rep. 2	Test Rep. 3	CTC
-2	NT	CT	CT	CT	CT
-3	NT	++++	++++	++++	0000
-4	++++	0+++	++++	++++	0000
-5	++++	0000	0000	0000	NT
-6	++++	0000	0000	0000	NT
-7	+0++	0000	0000	0000	NT
-8	0000	NT	NT	NT	NT
$TCID_{50}$/mL (log_{10})	7.25	4.25	4.50	4.50	2.50
Log_{10} Reduction	N/A	3.00	2.75	2.75	N/A
Percent Reduction	N/A	99.90%	99.82%	99.82%	N/A

Table S5. 4-hour challenge: Murine norovirus. "+" indicates virus-infected cells were present, while "0" indicates virus-infected cells were not detected. CT indicates concentration at which cytotoxicity was present. NT indicates "not tested". NC is the neutralizer control. NTC is the neutralizer cytotoxicity control. CTC is the cytotoxicity control.

Dilution (-Log10)	Virus Control	Test Rep. 1	Test Rep. 2	Test Rep. 3	NC	NTC	CTC
-2	NT	CT	CT	CT	NT	NT	CT
-3	NT	0000	0000	0000	++++	++++	0000
-4	++++	0000	0000	0000	++++	++++	0000
-5	++++	0000	0000	0000	++++	++++	NT
-6	++++	0000	0000	0000	++++	++++	NT
-7	++00	0000	0000	0000	000+	++0+	NT
-8	0000	NT	NT	NT	000+	0000	NT
$TCID_{50}$/mL (log_{10})	7.00	≤2.50	≤2.50	≤2.50	7.00	7.25	2.50
Log_{10} Reduction	N/A	≥4.50	≥4.50	≥4.50	N/A	N/A	N/A
Percent Reduction	N/A	>99.99%	>99.99%	>99.99%	N/A	N/A	N/A

Table S6. 24-hour challenge: Murine norovirus. "+" indicates virus-infected cells were present (n=4 per control or test replicate), while "0" indicates virus-infected cells were not detected. CT indicates concentration at which cytotoxicity was present. NT indicates "not tested". NC is the neutralizer control. NTC is the neutralizer cytotoxicity control. CTC is the cytotoxicity control.

Dilution (-Log10)	Virus Control	Test Rep. 1	Test Rep. 2	Test Rep. 3	NC	NTC	CTC
-2	NT	CT	CT	CT	NT	NT	CT
-3	NT	0000	0000	0000	++++	++++	0000
-4	++++	0000	0000	0000	++++	++++	0000
-5	++++	0000	0000	0000	++++	++++	NT
-6	++++	0000	0000	0000	++++	++++	NT
-7	0+00	0000	0000	0000	0+0+	0++0	NT
-8	+0+0	NT	NT	NT	0000	0000	NT
TCID50/mL (log10)	7.25	≤2.50	≤2.50	≤2.50	7.00	7.00	2.50
Log10 Reduction	N/A	≥4.75	≥4.75	≥4.75	N/A	N/A	N/A
Percent Reduction	N/A	>99.99%	>99.99%	>99.99%	N/A	N/A	N/A

Table S7. 30-minute challenge: Poliovirus type 1. "+" indicates virus-infected cells were present, while "0" indicates virus-infected cells were not detected. NT indicates "not tested". CTC is the cytotoxicity control.

Dilution (-Log10)	Virus Control	Test Rep. 1	Test Rep. 2	Test Rep. 3	CTC
-2	NT	++++	++++	++++	0000
-3	NT	++++	++++	++++	0000
-4	++++	++++	++++	++++	0000
-5	++++	++++	++++	++++	NT
-6	++++	++++	++++	++++	NT
-7	++++	++++	++++	++++	NT
-8	+++0	NT	NT	NT	NT
TCID50/mL (log10)	8.25	≥7.50	≥7.50	≥7.50	1.50
Log10 Reduction	N/A	≤0.75	≤0.75	≤0.75	N/A
Percent Reduction	N/A	≤82.22%	≤82.22%	≤82.22%	N/A

Table S8. 4-hour challenge: Poliovirus type 1. "+" indicates virus-infected cells were present, while "0" indicates virus-infected cells were not detected. NT indicates "not tested". NC is the neutralizer control. NTC is the neutralizer cytotoxicity control. CTC is the cytotoxicity control.

Dilution (-Log10)	Virus Control	Test Rep. 1	Test Rep. 2	Test Rep. 3	NC	NTC	CTC
-2	NT	++++	++++	++++	NT	NT	0000
-3	NT	++++	++++	++++	++++	++++	0000
-4	++++	++++	++++	++++	++++	++++	0000
-5	++++	++++	++++	++++	++++	++++	NT
-6	++++	++++	++++	++++	++++	++++	NT
-7	++++	++++	++++	++++	++++	++++	NT
-8	+0++	NT	NT	NT	+0+0	0+00	NT
TCID50/mL (log10)	8.25	≥7.50	≥7.50	≥7.50	8.00	7.75	1.50
Log10 Reduction	N/A	≤0.75	≤0.75	≤0.75	N/A	N/A	N/A
Percent Reduction	N/A	≤82.22%	≤82.22%	≤82.22%	N/A	N/A	N/A

Table S9. 24-hour challenge: Poliovirus type 1. "+" indicates virus-infected cells were present (n=4 per control or test replicate), while "0" indicates virus-infected cells were not detected. NT indicates "not tested". NC is the neutralizer control. NTC is the neutralizer cytotoxicity control. CTC is the cytotoxicity control.

Dilution (-Log$_{10}$)	Virus Control	Test Rep. 1	Test Rep. 2	Test Rep. 3	NC	NTC	CTC
-2	NT	++++	++++	++++	NT	NT	0000
-3	NT	++++	++++	++++	++++	++++	0000
-4	++++	++++	++++	++++	++++	++++	0000
-5	++++	++++	++++	++++	++++	++++	NT
-6	++++	++++	++++	++++	++++	++++	NT
-7	++++	++++	++++	++++	++++	++++	NT
-8	00++	NT	NT	NT	++00	+++0	NT
TCID$_{50}$/mL (log$_{10}$)	8.00	≥7.50	≥7.50	≥7.50	8.00	8.25	1.50
Log$_{10}$ Reduction	N/A	≤0.50	≤0.50	≤0.50	N/A	N/A	N/A
Percent Reduction	N/A	≤68.38%	≤68.38%	≤68.38%	N/A	N/A	N/A

Table S10. 30-minute challenge: SARS-CoV-2 (original). "+" indicates virus-infected cells were present, while "0" indicates virus-infected cells were not detected. NT indicates "not tested". CTC is the cytotoxicity control.

Dilution (-Log$_{10}$)	Virus Control	Test Rep. 1	Test Rep. 2	Test Rep. 3	CTC
-2	NT	CT	CT	CT	CT
-3	++++	++++	0+++	++0+	0000
-4	++++	+++0	++00	++++	0000
-5	++++	0000	0000	0000	NT
-6	+00+	0000	0000	0000	NT
-7	0000	0000	0000	0000	NT
TCID$_{50}$/mL (log$_{10}$)	6.00	4.25	3.75	4.25	2.50
Log$_{10}$ Reduction	N/A	1.75	2.25	1.75	N/A
Percent Reduction	N/A	98.22%	99.44%	98.22%	N/A

Table S11. 4-hour challenge: SARS-CoV-2 (original). "+" indicates virus-infected cells were present, while "0" indicates virus-infected cells were not detected. NT indicates "not tested". NC is the neutralizer control. NTC is the neutralizer cytotoxicity control. CTC is the cytotoxicity control.

Dilution (-Log$_{10}$)	Virus Control	Test Rep. 1	Test Rep. 2	Test Rep. 3	NC	NTC	CTC
-2	NT	CT	CT	CT	NT	NT	CT
-3	++++	0000	00+0	0000	++++	++++	0000
-4	++++	0000	0000	0000	++++	++++	0000
-5	++++	0000	0000	0000	++++	+++0	NT
-6	00+0	0000	0000	0000	0000	00+0	NT
-7	0000	0000	0000	0000	0000	0000	NT
TCID$_{50}$/mL (log$_{10}$)	5.75	≤2.50	2.75	≤2.50	5.50	5.50	2.50
Log$_{10}$ Reduction	N/A	≥3.25	3.00	≥3.25	N/A	N/A	N/A
Percent Reduction	N/A	≥99.94%	99.90%	≥99.94%	N/A	N/A	N/A

Table S12. 24-hour challenge: SARS-CoV-2 (original). "+" indicates virus-infected cells were present, while "0" indicates virus-infected cells were not detected. CT indicates concentration at which cytotoxicity was present. NT indicates "not tested". NC is the neutralizer control. NTC is the neutralizer cytotoxicity control. CTC is the cytotoxicity control.

**Dilution (-Log$_{10}$)**	**Virus Control**	**Test Rep. 1**	**Test Rep. 2**	**Test Rep. 3**	**NC**	**NTC**	**CTC**
-2	NT	CT	CT	CT	NT	NT	CT
-3	++++	0000	0000	0000	++++	++++	0000
-4	++++	0000	0000	0000	++++	++++	0000
-5	++++	0000	0000	0000	++00	+++0	NT
-6	0000	0000	0000	0000	0000	0000	NT
-7	0000	0000	0000	0000	0000	0000	NT
TCID$_{50}$/mL (log$_{10}$)	5.50	≤2.50	≤2.50	≤2.50	5.00	5.25	2.50
Log$_{10}$ Reduction	N/A	≥3.00	≥3.00	≥3.00	N/A	N/A	N/A
Percent Reduction	N/A	≥99.90%	≥99.90%	≥99.90%	N/A	N/A	N/A

Table S13. 30-minute challenge: SARS-CoV-2 (omicron). "+" indicates virus-infected cells were present, while "0" indicates virus-infected cells were not detected. NT indicates "not tested". CTC is the cytotoxicity control.

**Dilution (-Log$_{10}$)**	**Virus Control**	**Test Rep. 1**	**Test Rep. 2**	**Test Rep. 3**	**CTC**
-2	NT	CT	CT	00++	CT
-3	++++	0000	++00	0000	0000
-4	++++	0000	0000	0000	0000
-5	++++	0000	0000	0000	NT
-6	00+0	0000	0000	0000	NT
-7	0000	0000	0000	0000	NT
TCID$_{50}$/mL (log$_{10}$)	5.75	≤2.50	3.00	3.00	2.50
Log$_{10}$ Reduction	N/A	≥3.25	2.75	2.75	N/A
Percent Reduction	N/A	≥99.94%	99.82%	99.82%	N/A

Table S14. 4-hour challenge: SARS-CoV-2 (omicron). "+" indicates virus-infected cells were present, while "0" indicates virus-infected cells were not detected. NT indicates "not tested". NC is the neutralizer control. NTC is the neutralizer cytotoxicity control. CTC is the cytotoxicity control.

**Dilution (-Log$_{10}$)**	**Virus Control**	**Test Rep. 1**	**Test Rep. 2**	**Test Rep. 3**	**NC**	**NTC**	**CTC**
-2	NT	CT	CT	CT	NT	NT	CT
-3	++++	0000	0000	0000	++++	++++	0000
-4	++++	0000	0000	0000	++++	++++	0000
-5	++++	0000	0000	0000	+0++	++++	NT
-6	+000	0000	0000	0000	0000	0000	NT
-7	0000	0000	0000	0000	0000	0000	NT
TCID$_{50}$/mL (log$_{10}$)	5.75	≤2.50	≤2.50	≤2.50	5.25	5.50	2.50
Log$_{10}$ Reduction	N/A	≥3.25	≥3.25	≥3.25	N/A	N/A	N/A
Percent Reduction	N/A	≥99.94%	≥99.94%	≥99.94%	N/A	N/A	N/A

Table S15. 24-hour challenge: SARS-CoV-2 (omicron). "+" indicates virus-infected cells were present, while "0" indicates virus-infected cells were not detected. CT indicates concentration at which cytotoxicity was present. NT indicates "not tested". NC is the neutralizer control. NTC is the neutralizer cytotoxicity control. CTC is the cytotoxicity control.

**Dilution (-Log$_{10}$)**	**Virus Control**	**Test Rep. 1**	**Test Rep. 2**	**Test Rep. 3**	**NC**	**NTC**	**CTC**
-2	NT	CT	CT	CT	NT	NT	CT
-3	++++	0000	0000	0000	++++	++++	0000
-4	++++	0000	0000	0000	++++	++++	0000
-5	++++	0000	0000	0000	0+++	++0+	NT
-6	0000	0000	0000	0000	0000	0000	NT
-7	0000	0000	0000	0000	0000	0000	NT
TCID$_{50}$/mL (log$_{10}$)	5.50	≤2.50	≤2.50	≤2.50	5.25	5.25	2.50
Log$_{10}$ Reduction	N/A	≥3.00	≥3.00	≥3.00	N/A	N/A	N/A
Percent Reduction	N/A	≥99.90%	≥99.90%	≥99.90%	N/A	N/A	N/A
